# Validity and Reliability of Dysphagia Outcome Severity Scale (DOSS) When Used to Rate Flexible Endoscopic Evaluations of Swallowing (FEES)

**DOI:** 10.1007/s00455-024-10732-z

**Published:** 2024-07-24

**Authors:** Johanna Fransson, Sofia Thorén, Jenny Selg, Liza Bergström, Patricia Hägglund

**Affiliations:** 1 Speech and Language Pathology, Department of Clinical Sciences, Faculty of Medicine, Umeå University, 90187 Umeå, Sweden; 2 Division of Neurology, Department of Clinical Sciences, Karolinska Institute, Danderyd University Hospital, Stockholm, Sweden; 3 Remeo Stockholm, Torsten Levenstams Väg 8, Sköndal, Stockholm, Sweden

**Keywords:** Oropharyngeal dysphagia, Deglutition, Assessment, Functioning, ICF

## Abstract

The Dysphagia Outcome and Severity Scale is used both clinically and within dysphagia research, internationally. Although it was developed using videofluoroscopic swallowing studies, it is frequently used to rate Flexible Endoscopic Evaluations of Swallowing. The validity and reliability of DOSS-use with FEES, however, has not previously been evaluated. This study investigated the validity and rater reliability of clinicians using DOSS to rate FEES. Eleven Speech-Language Pathologists (SLPs) with varied dysphagia experience were recruited to review and DOSS-rate 17 soundless FEES (198 bolus swallows) recorded from 11 heterogenic dysphagic patients (2 cases with repeat FEES) and 4 healthy adults. The SLPs DOSS-ratings were compared against the initial comprehensive dysphagia evaluation (including patient diagnosis, interview, cranial nerve and complete FEES assessment) with Functional Oral Intake Scale (FOIS) and DOSS outcome measures. The SLPs were blinded to patient details and comprehensive dysphagia examination. Re-randomised rating of FEES cases occurred two weeks later (intra rater reliability). Criterion validity for DOSS-ratings (compared against comprehensive dysphagia evaluation with FOIS and DOSS) were strong–very strong (*r*_s _= 0.858 and 0.936 respectively; *p* < 0.001). Inter rater reliability demonstrated high agreement (α = 0.891), also intra rater reliability demonstrated almost perfect agreement (Kw = 0.945). This study’s results, with strong–very strong criterion validity and high rater reliability by SLPs, adds to the evidence for DOSS-use with FEES. Future validity research comparing DOSS with both FEES and VFSS simultaneously is recommended.

**Supplementary Information:**

The online version contains supplementary material available at 10.1007/s00455-024-10732-z.

## Introduction

Dysphagia is prevalent across a range of adult populations. This includes but is not limited to dysphagia within head and neck cancer, stroke, head injuries, high spinal cord injuries, neurological disorders such as Guillain Barré syndrome, Parkinson’s disease, motor neuron disease, and the intensive care/tracheostomised population [1–5]. The impact of dysphagia among the elderly (presbyphagia), sarcopenic dysphagia and within aged care facilities has also been highlighted in recent years [6, 7]. Consequences of dysphagia are significant and may include malnutrition, dehydration, aspiration pneumonia, and premature death [8–11]. Apart from clinical impacts, dysphagia can negatively affect a person’s everyday life with research identifying reduced quality of life and reduced socialisation around meal-times (an important component of many societies today) [12]. Furthermore, within hospital and other healthcare settings, dysphagia is associated with longer lengths-of-stay, healthcare costs and poorer patient outcomes [1, 13–15].

Given the negative impact of dysphagia on patient and healthcare outcomes, it is important that dysphagia is optimally managed. In the field of dysphagia, gold standard assessment methods include (1) Flexible Endoscopic Evaluation of Swallowing (FEES) and (2) Videofluoroscopic Swallowing Study (VFSS) [16–19]. Several different rating scales are used to assess features of the swallow process during FEES and VFSS and include the Penetration-Aspiration Scale (PAS) [20], the Normalized Residue Ratio Scale (NRRS) [21], the Yale Pharyngeal Residue Severity Rating Scale (YPRSRS) [22], the New Zealand Secretion Scale (NZSS) [23] and more recently the Visual Analysis of Swallowing Efficiency and Safety (VASES) [24]. These scales investigate specific components of the swallow, such as airway safety (penetration, aspiration) before, during and after the swallow (of saliva, food/drink), pharyngeal residue and efficiency of the swallow. Of note, the Penetration-Aspiration Scale was developed and validated based on VFSS yet is extensively used with FEES both clinically and with research [25, 26].

These physiological impairment-based scales are important for standardisation of dysphagia assessment. However, they lack a holistic and person-centred dysphagia management perspective as per the World Health Organisation’s (WHO) International Classification of Functioning, Disability and Health (ICF). The ICF framework considers not only medical diagnosis and assessment at a physiological impairment level but advocates for assessment and management further incorporating the impact on functional activity, and the patient’s participation success, while also considering environmental and personal factors [27].

Few dysphagia assessment measures consider the WHO’s ICF perspective. The Dysphagia Outcome and Severity Scale (DOSS) [28] was developed, validated and is one of the few rating scales to somewhat address the lack of holistic/ICF aspects when rating dysphagia severity. The DOSS considers, not only swallow impairment at the physiological level, but also functional and activity aspects, as per ICF recommendations. With the DOSS, the patient’s swallowing is assessed using seven severity levels (1–7), from severe dysphagia to normal swallowing function. The severity scale includes aspects of swallow safety and efficiency, considering the oral and pharyngeal phases, bolus transport, airway protection and pharyngeal retention. Apart from these physiological evaluation parameters, the DOSS incorporates functional impacts such as nutritional intake requirements, range of diet, and level of support/independence in eating and drinking; where severity level 1–2 indicates non-oral nutrition requirements, 3–5 indicates full per oral (PO) nutrition with a modified diet, strategies and/or different levels of support/dependence, and 6–7 indicates full PO, normal diet, with or without additional strategies employed dependently.

It is important to note that the DOSS was developed and validated based on VFSS [28], with different research groups consequently using the DOSS to further validate other rating scales, often demonstrating strong and/or significant correlations [29–31]. Very high DOSS-rater reliability has been reported by some authors [28, 32] yet not by others [33]. Although the DOSS was validated using VFSS, it is widely used as an assessment outcome measure for FEES [30, 34, 35], though this practice is criticised by many clinicians and researchers alike. Consequently, this practice of using the DOSS to rate FEES warrants further evaluation, particularly in terms of psychometric properties (validity and reliability). Until recently, an overall dysphagia severity outcome scale has not been available in Sweden [36]. Given the strong validity and reliability of the DOSS in the majority of publications, and the holistic patient management aspects considering some ICF components, a translation of the DOSS into Swedish (DOSS-S) was recently validated [36]. The DOSS-S was found to have high validity (Content Validity Index = 0.84–0.99; criterion validity, *r* = 0.89, p < 0.01). Rater reliability of DOSS-S was also assessed (n = 18 SLPs) using the International Dysphagia Diet Standardisation Initiative—Functional Diet Scale (IDDSI-FDS) cases [37], resulting in very high inter and intra rater reliabilities (ICC > 0.90).

With the above in mind and given that FEES is the most widely used instrumental dysphagia assessment tool in Sweden, the need to validate and evaluate clinician reliability using DOSS with FEES, rather than VFSS, is evident.

### Aims

Consequently, the aim of the present study was to investigate the validity and reliability of clinicians using DOSS-S to rate dysphagia assessments via FEES.

### Research questions

1. How valid is the DOSS-S when used by a range of experienced clinicians to rate FEES of heterogenic dysphagic and non-dysphagic patients?
2. How reliable (inter and intra rater reliability) are clinicians in their DOSS-S ratings of FEES?

## Methods

### Study Design

This prospective validity and reliability study was approved by the Swedish Ethical Review Authority (Dnr: 2020–05246, 2020–04817 and 2022–06021–02) and conducted according to the Helsinki Declaration [38]. For quality reporting, this paper was written in accordance with the Guidelines for reporting reliability and agreement studies (GRRAS) [39], see appendix A.

## Participants: DOSS-S Raters

Eleven Speech-Language Pathologists (SLPs) were recruited to rate 17 FEES (198 bolus swallows) using the DOSS-S. The SLPs were recruited via convenience sampling with inclusion criteria: (i) registered SLP, (ii) experience of working with dysphagia, and (iii) competence to perform and assess FEES. A range of dysphagia experience (in years), and FEES experience was considered during recruitment. This dysphagia experience data was collected, along with self-reported experience of DOSS or DOSS-S use (on a scale of 1–4, where 1 = never used DOSS, 2 = used DOSS a few times, 3 = used DOSS from time to time, 4 = used DOSS frequently). See Table 1 for SLP participant details.

**Table 1:** Demographic Data for the Speech-Language Pathologists (raters)

Rater	Region	Dysphagia Experience in years		FEES Experience in years		Self-rated DOSS Experience
		M	SD	M	SD	
The entire group (*n* = 11)		9.25	6.04	7.11	4.1	
1	Götaland	13		6		None
2	Svealand	8		6		Moderate
3	Norrland	22		15		Little
4	Svealand	13		10		Little
5	Götaland	11		10		Moderate
6	Götaland	3		3		Little
7	Götaland	12.5		11		A lot
8	Norrland	4.75		4.75		Moderate
9	Norrland	1.5		1.5		Moderate
10	Norrland	3		3		A lot
11	Norrland	10		8		A lot

*FEES* Flexible endoscopic evaluation of swallowing, *DOSS* Dysphagia Outcome and Severity Scale, *M* Mean, *SD* Standard deviation

## Participants: Providing FEES

To ensure a representative range across the DOSS severity levels (1–7), 17 selected FEES were collected from the research teams’ collective bank of research subject recordings and edited, providing a total of 198 bolus swallows (*mean* = 11.65 bolus swallows/FEES). The number of FEES (17) were selected via convenience sampling to ensure a range of FEES and feasibility for volunteer raters (total FEES viewing time = 99 min). The FEES were from 11 heterogenic dysphagic patients, with two patients providing two separate FEES, 2–3 weeks apart, and four healthy adults (Table 2).

**Table 2:** Demographic data from dysphagic patients and adults (n = 15) who underwent the comprehensive FEES (gold standard) assessment

FEES (n = 17)	Age	Medical diagnosis and history	DOSS	FOIS
1	57	High spinal cord injury. Weaned from ventilator. Tracheostomy with cuff deflated, speaking valve insitu at time of FEES	1	1
2	61	Aneurysm, coiled, subarachnoid haemorrhage. Hemi-craniectomy, intracerebral pressure instability, shunt. Tracheostomy with cuff deflated, speaking valve insitu at time of FEES	1	1
3	71	Multi-trauma, prolonged intubation	1	1
4	62	High Spinal cord injury, trauma. Treated with Anterior Cervical Spine Surgery (ACSS) approach. Tracheostomy and on ventilator cuff inflated, during FEES	2	2
5	77	Brainstem stroke	2	3
6	32	High Spinal cord injury, trauma. Treated with ACSS approach. Weaned from ventilator. Tracheostomy with cuff deflated, speaking valve insitu at time of FEES	3	5
7	42	High Spinal cord injury, spinal stenosis. Treated with ACSS approach	3	5
*8*	*57*	*Patient 1, repeat FEES*—see above for medical diagnosis/history. New FEES, 3 weeks later, tracheostomy with cuff deflated, capped	3	5
*9*	*61*	*Patient 2, repeat FEES*—see above for medical diagnosis/history. New FEES, 3 weeks later, without tracheostomy (decannulated)	4	6
10	72	Ischemic stroke	4	6
11	64	Brain tumour bleed, enlarged lateral and third ventricles, compression of fourth ventricle, oedema. Tracheostomy with cuff deflated, speaking valve insitu at time of FEES	5	3
12	74	High Spinal cord injury, spinal stenosis. Treated with ACSS approach	5	7
13	43	Healthy person	6	7
14	48	Healthy person	6	7
15	25	Healthy person	7	7
16	25	Healthy person	7	7
17	58	High Spinal cord injury, spinal stenosis. Treated with ACSS approach	7	7

*FEES* Flexible endoscopic evaluation of swallowing. *DOSS* Dysphagia Outcome and Severity Scale, *FOIS* Functional Oral Intake Scale. Of the 15 participants, 9 were male, 6 were female*.* Two patients with repeat FEES = indicated with* italics*

Evaluation of validity and reliability was achieved through the following three procedural steps. Step 1: Collection of data from a gold standard comprehensive dysphagia evaluation with FEES (reference test), assessed with FOIS and DOSS scores—for validity testing. Step 2: Compilation of FEES recordings to be sent to a range of DOSS-S raters. Step 3: The DOSS-S rating procedure for inter and intra rater reliability and validity testing.

## Step 1: Comprehensive Dysphagia Evaluation with FEES

The 15 adults (providing a total of 17 FEES) underwent a comprehensive ‘gold standard’ dysphagia evaluation, which included (a) medical diagnosis and history, (b) patient interview, when appropriate, (c) cranial nerve and oromotor assessment, (d) complete FEES assessment—with sound and the entire film period (recording also the entire between-bolus periods), and (e) discussion with the patient regarding assessment results and recommendations, i.e., patient participation and inclusion for collaborative dysphagia management planning (for increased ICF perspective/information). A standard FEES protocol based on Langmore [40] was followed including different bolus consistencies, as per IDDSI, and volumes. The FEES procedure included IDDSI 0, 2, 4, 6, 7 given as 3 × 5 mL and 3 × 10 mL. IDDSI 0 also included continual mouthfuls. The FEES protocol was followed as far as possible, without compromising patient status/health. Where patient safety risks (i.e., significant aspiration or risk for delayed aspiration due to significant residue, plus poor sensation and motor ability to clear pharyngeal/laryngeal residue) were observed, then the FEES in terms of number of boluses, volumes and consistencies was modified/ceased.

This comprehensive dysphagia evaluation (gold standard) as described above, was conducted by a total of four different clinicians with 3–24 years dysphagia experience (*mean* = 11.25 years) and with 1–18 years FEES experience (*mean* = 8.5 years). These clinicians worked in pairs where one performed the FEES and one assisted during the procedure. Each pair included a clinician with extensive knowledge and experience with DOSS (see previous work presented within article by Movander et al., 2023 [36]). The dysphagia evaluation incorporated both oral and pharyngeal (FEES) aspects along with ICF considerations (function and structure, activities and participation, as well as environmental patient/personal factors) in the evaluation and dysphagia management planning. Following this comprehensive evaluation and patient participation in management planning, the Functional Oral Intake Scale (FOIS) [41] scores and DOSS-S scores were determined, and recommendations made. Both the FOIS and DOSS-S scores were assigned by the clinicians performing the comprehensive gold standard dysphagia evaluation, as described above, and was based on the overall dysphagia assessment and patient information.

## Step 2: Compilation of FEES Recordings for DOSS-S Raters

Prior to sending the FEES recordings to the 11 SLP raters, the 17 FEES with a total of 198 swallows, were edited in order to remove sound (i.e., identifiable patient voices) and excessive information such as time in-between boluses without pharyngeal/laryngeal activity. The edited FEES (total = 198 bolus swallows, *mean* = 11.65 boluses/FEES) varied in the number of consistencies assessed, depending on aspiration/risk for aspiration, and ranged from 2.04 to 12.06 min. The total running time of the 17 edited FEES was 99.36 min.

## Step 3: DOSS-S Ratings

The SLP clinicians independently rated the above 17 edited FEES using the DOSS-S, twice, via an online secured platform (Canvas) as used by Swedish universities, requiring multifactorial authentication. The raters were blinded to the FEES protocol (order and number of bolus presentations), patient information and all other comprehensive dysphagia evaluation information (as per Step 1). Raters were given access to the DOSS-S rating scale one week prior to rating. When rating the FEES, the clinicians had the opportunity to view each patient’s FEES several times, pause, rewind, and change the playback speed, after which the clinicians then assigned a DOSS-S level to that patient. To evaluate intra rater reliability the SLP clinicians performed a second DOSS-S rating of the same FEES (in a re-randomised order) two weeks post first rating.

## Statistical Analysis

All statistical analyses were performed with IBM/SPSS Statistics (version 29). For convergent validity (the correlation between the FOIS and DOSS rating scales used at the time of the comprehensive dysphagia evaluation with FEES), Spearmans’s rank correlation coefficient was employed. Additionally, criterion validity between the 11 clinicians DOSS-S ratings of the 17 edited, soundless FEES and the initial FOIS and DOSS-S ratings determined at the time of the comprehensive dysphagia (gold standard FEES) evaluation was also calculated using Spearman’s rank correlation coefficient. The calculation was conducted using the median of the clinicians DOSS-S ratings.

Inter rater reliability, analysed using the first round of ratings, was conducted using the Krippendorff’s alpha [42]. Intra rater reliability was calculated using weighted Kappa with quadratic weights [43]. Descriptive analyses of Percent Exact Agreement (PEA) between raters and Percent Close Agreement (PCA: one point difference) were also calculated.

For interpretation of statistical analyses, a Spearman’s correlation coefficient of 0.21–0.35 = weak correlation, 0.36–0.67 = moderate, 0.68–0.90 = strong and 0.91–1.00 = very strong correlation [44]. Inter rater reliability interpretation for Krippendorff alpha values of > 0.80 = acceptable level of reliability and 1.0 = perfect reliability [42]. A Kappa value between 0.00–0.20, is considered no agreement, 0.21–0.39 minimal, 0.40–0.59 weak, 0.60–0.79 moderate, 0.80–0.90 strong and > 0.90 almost perfect [45].

## Results

### Participants

Eleven SLP’s representing all 3 regions in Sweden were recruited to rate the 17 FEES using DOSS-S (see Table 1). Raters had between 1.5–22 years of clinical dysphagia experience, and 1.5–15 years of FEES experience. For intra rater reliability, 10/11 SLP’s were available for the re-rating; thus, one drop-out occurred.

## Validity

Firstly, in terms of convergent validity, the DOSS outcome from the initial gold standard, comprehensive dysphagia evaluation (with patient diagnosis, background, complete FEES etc.), when compared with FOIS, showed very strong correlation (*r*_s_ = 0.924, *p* < 0.001).

**Fig. 1:**
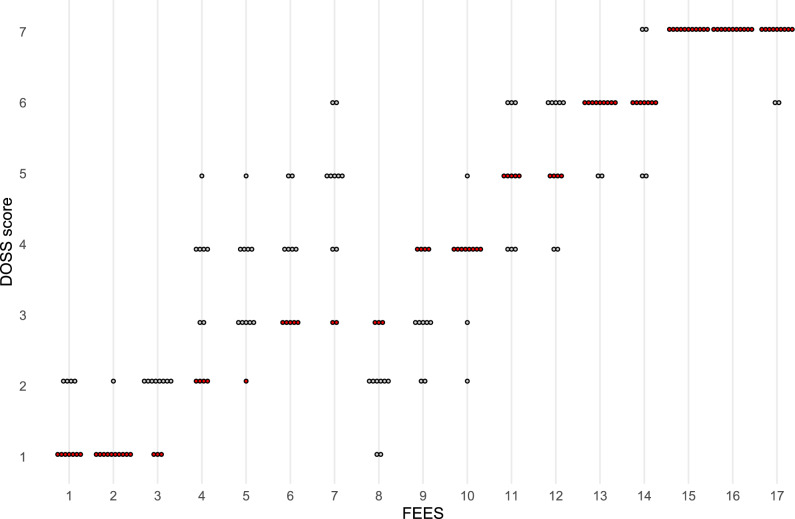
DOSS-S ratings for FEES with corresponding comprehensive dysphagia evaluation DOSS-score. The red markings indicate the original DOSS score from the comprehensive dysphagia evaluation, while each dot represents one of 11 SLP’s DOSS ratings

Secondly, the median DOSS-S ratings (by SLP raters using edited FEES without sound), when compared against the initial comprehensive dysphagia evaluations FOIS and DOSS-S scores, demonstrated strong–very strong criterion validity (*r*_s_ = 0.858 p < 0.001 and *r*_s_ = 0.936 p < 0.001) respectively. For complete overview of SLPs ratings and comprehensive dysphagia evaluations for each FEES, see Fig. 1.

## Inter Rater Reliability

The overall inter rater reliability between the SLP’s (*n* = 11) demonstrated high agreement (α = 0.891, 95% CI = 0.879–0.902). Descriptive statistics (Table 3) revealed that the majority of the SLP’s had the same ratings for the 17 patient FEES. Percentage exact agreement and percent close agreement are shown in Table 3.

**Table 3:** SLP DOSS-S ratings for each of the 17 cases

FEES	DOSS-S ratings							PEA	PCA
	1	2	3	4	5	6	7		
1	7	4	–	–	−	−	−	63.6	100
2	10	1	−	−	−	−	−	90.9	100
3	3	8	−	−	−	−	−	72.7	100
4	−	4	2	4	1	−	−	36.4	63.7
5	−	1	5	4	1	−	−	45.5	90.9
6	−	−	5	4	2	−	−	45.5	81.8
7	−	−	2	2	5	2	−	45.5	81.8
8	2	6	3	−	−	−	−	54.5	100
9	−	2	5	4	−	−	−	45.5	100
10	−	1	1	8	1	−	−	72.7	90.9
11	−	−	−	3	5	3	−	45.5	100
12	−	−	−	2	4	5	−	45.5	81.8
13	−	−	−	−	2	9	−	81.8	100
14	−	−	−	−	2	7	2	63.6	100
15	−	−	−	−	−	−	11	100	100
16	−	−	−	−	−	−	11	100	100
17	−	−	−	−	−	2	9	81.8	100

*SLP* Speech Language Pathologist, *FEES* Flexible Endoscopic Evaluation of Swallowing, *PEA* Percent Exact Agreement, *PCA* Percent Close Agreement = 1 point difference

## Intra Rater Reliability

Intra rater reliability for the overall group (*n* = 10) indicated almost perfect agreement (*K*w = 0.945). The individual intra rater reliability for the 10 raters also indicated strong–almost perfect agreement (Kw range = 0.884–0.993), see Table 4.

**Table 4:** SLPs’ intra rater reliability when rating with DOSS-S

SLP (*n* = 10)	Weighted Kappa	95% CI	Agreement with FEES (*n* = 17)	
			PEA	PCA
Group (*n* = 10)	0.945	0.924–0.967	72.4	95.3
1	0.921	0.832–1.010	76.5	82.4
2	0.911	0.842–0.980	47.1	94.1
3	0.993	0.979–1.007	94.1	100
4	0.954	0.919–0.990	64.7	100
5	0.987	0.969–1.005	88.2	100
7	0.884	0.744–1.023	58.8	94.1
8	0.946	0.884–1.009	76.5	94.1
9	0.934	0.865–1.002	64.7	94.1
10	0.924	0.843–1.005	64.7	94.1
11	0.984	0.962–1.007	88.2	100

*SLP* Speech-Language Pathologist, *CI* Confidence Interval, *FEES* Flexible endoscopic evaluation of swallowing, *PEA* Percent Exact Agreement, *PCA* Percent Close Agreement = 1 point difference

## Discussion

This study investigated the validity and reliability of clinicians using DOSS-S to rate dysphagia via FEES. The clinicians showed a rage regarding dysphagia experience (1, 5–22 years) and experience using DOSS. Results demonstrate significant results (*p* < 0.001) and strong–very strong criterion validity when comparing SLP clinician DOSS-S ratings with gold standard FOIS (*r*_s_ = 0.858) and DOSS-S (*r*_s_ = 0.936). Both inter rater and intra rater reliability demonstrated high agreement (α = 0.891; Kw = 0.945). These results will be further discussed below in relation to previous literature, clinical application, and future research recommendations.

In terms of validity, the current study compared the DOSS-S outcome with the FOIS outcome, both scored from a comprehensive dysphagia evaluation including FEES (gold standard). The FOIS has previously been shown to be a valid and reliable assessment scale for both FEES [46] and VFSS [47] and, furthermore, correlates significantly with DOSS scores [48]. In the current study, (i) the very strong convergent validity between DOSS and FOIS following the initial gold standard comprehensive dysphagia evaluation, and also (ii) the strong criterion validity between the blinded SLPs median DOSS-S ratings of edited FEES and the FOIS ratings, provides promising evidence for clinical application of using DOSS to rate FEES.

The strong validity results for this study are particularly of interest considering the different conditions for the SLP DOSS-S raters using voiceless, edited FEES (and blinded to patient diagnosis and extensive patient information), versus the original FOIS and DOSS-S ratings from the comprehensive dysphagia evaluation (gold standard), which included (a) medical diagnosis and history, (b) patient interview, when appropriate, (c) cranial nerve and oromotor assessment, (d) a complete FEES assessment with sound and the entire film period (with the between bolus period recorded), and (e) discussion with the patient regarding assessment results and recommendations. Given the lack of information available to the SLP DOSS-S raters, versus the comprehensive dysphagia evaluation condition, the high criterion validity is noteworthy and discussed further below.

In terms of rater reliability, results from the current study demonstrated high *inter* rater reliability (α = 0.891), findings which are similar to the original DOSS study by O’Neil et al. [28] (90% agreement), and Kidney et al. [32] (92% agreement, *K*w = 0.67) yet is in contrast to the study by Zarkada and Regan [33] with fair rater agreement (κ = 0.36). Similarly, *intra* rater reliability results for the current study were almost perfect (*K*w = 0.945), which is congruent with other research results (> 93% agreement and high kappas, *K*w = 0.89) [28, 32]. The divergent results between studies are likely due to methodological differences. O’Neil et al. [28] evaluated rater reliability of four DOSS-trained SLPs based on 135 written VFSS reports which included (a) a full patient medical history, (b) patient level of alertness, cognitive status and ability to follow commands, (c) the patient’s current diet, (d) oro-motor and voice assessment, and (e) a detailed oral and pharyngeal phase description from the VFSS. In contrast, the study by Zarkada and Regan [33] used the following methodology: 17 (experienced versus minimally experienced) SLPs who rated eight VFSS clips, of which five included sound. Results indicated overall fair reliability (κ = 0.36), however, significantly greater inter rater reliability for (i) experienced versus minimal experienced clinicians, and (ii) VFSS clip ratings with sound versus those without sound.

The incongruency between the above study results may be further explained by considering that the DOSS incorporates several holistic, person-centered and ICF aspects (not just instrumental assessment, impairment-based information) but also a person’s activities, participation, environmental and personal factors, therefore perhaps the greater holistic information from the written VFSS reports by O’Neill & colleagues [28] explains the greater reliability results versus if DOSS was rated using isolated VFSS clips only, as per Zarkada and Regan [33]. This explanation, although plausible, is however, not congruent with the current study’s results since the current study used edited FEES recordings only (excluding extensive patient information) and yet strong validity and high rater reliability was indeed demonstrated.

Reasons for such high validity and reliability results in the current study may include methodological considerations such as (a) that FEES allows for longer viewing time of pharyngeal responses (both motor and sensory) and patient management of residue, (b) laryngeal responses (even without sound, a cough and effective laryngeal/pharyngeal clearance of penetration/residue can be identified), and (c) that raters in the current study were allowed to view the FEES several times, pause, change the playback speed, and rewind – reflecting the clinical practice for assessments with FEES. Finally, the number of FEES rated (198 bolus swallows for 17 patients) may have also added to the current study’s strong results.

## Limitations and Future Directions

As with all research, this study has its limitations. The current study limited the SLP DOSS-S raters during their assessment of the edited (soundless) FEES by not providing the additional important patient information from the comprehensive gold standard dysphagia evaluation. Although blinding of the SLP DOSS raters was considered a methodological strength of the current study, this is not reflective of dysphagia practice and, in fact, negatively impacts the DOSS rating results in this study. Additionally, the FEES were soundless (due to patient de-identification requirements), which may have also impacted the DOSS-S ratings. To assign a DOSS-S level, the rater needs to consider whether the person coughs and clears the throat spontaneously or on request. As the FEES lacked sound, a possible verbal prompt could not be ascertained, and the rater was required to interpret and rate DOSS without this information. This lack of extra information, including verbal cuing and use of compensatory strategies, may have negatively affected the rater’s ability to thoroughly assess and assign a DOSS-S rating. The lack of sound also meant that some auditory-based clinical signs of penetration or aspiration were lost, such as voice changes [16, 40]. Further research should investigate the importance of sound, the influence of including patient background information and compensatory strategies, and this impact on DOSS-S ratings. The effect, if any, of rater experience on DOSS-S ratings and rater reliability could also be investigated in future studies. Finally, this study investigated convergent validity with FOIS only. Future research comparing DOSS with both FEES and VFSS simultaneously is warranted, as is perhaps comparison with rating scales such as PAS [20], YPRSRS) [22], and VASES [24].

## Conclusions

Results from this study indicate that the DOSS when used with FEES has strong–very strong convergent and criterion validity. High rater reliability was also demonstrated by clinicians (with varied dysphagia experience) who rated DOSS using FEES from a heterogenic dysphagic population. This study contributes to the evidence supporting use of DOSS as a valid and reliable rating scale to use with FEES.

## Supplementary Information

Below is the link to the electronic supplementary material.

Supplementary file1 (DOCX 27 KB)

## Data Availability

Data supporting the results and analyses are available on request from the final author (PH).
