# Supplementary material for: Validity and Reliability of Dysphagia Outcome Severity Scale (DOSS) When Used to Rate Flexible Endoscopic Evaluations of Swallowing (FEES)
Source: Dysphagia. 2024 Jul 24;40(2):343–52. doi: 10.1007/s00455-024-10732-z (PMC11893719; doi:10.1007/s00455-024-10732-z)
Supplement: Supplementary file 1 — Supplementary file1 (DOCX 27 KB) [file 455_2024_10732_MOESM1_ESM.docx]

GRRAS checklist for reporting of studies of reliability and agreement

Version based on Table I in: Kottner J, Audigé L, Brorson S, Donner A, Gajeweski BJ, Hróbjartsson A, Robersts C, Shoukri M, Streiner DL. Guidelines for reporting reliability and agreement studies (GRRAS) were proposed. J Clin Epidemiol. 2011;64(1):96-106

| **Section** | **Item #** | **Checklist item** | **Reported on page #** |
| --- | --- | --- | --- |
| Title/Abstract | 1 | Identify in title or abstract that interrater/intrarater reliability or agreement was investigated. | 1 |
| Introduction | 2 | Name and describe the diagnostic or measurement device of interest explicitly. | 3 |
|  | 3 | Specify the subject population of interest. | 2 |
|  | 4 | Specify the rater population of interest (if applicable). |  |
|  | 5 | Describe what is already known about reliability and agreement and provide a rationale for the study (if applicable). | 3, 4 |
| Methods | 6 | Explain how the sample size was chosen. State the determined number of raters, subjects/objects, and replicate observations. | 5, 6,  8 |
|  | 7 | Describe the sampling method. | 5, 6 |
|  | 8 | Describe the measurement/rating process (e.g. time interval between repeated measurements, availability of clinical information, blinding). | 8 |
|  | 9 | State whether measurements/ratings were conducted independently. | 8 |
|  | 10 | Describe the statistical analysis. | 8, 9 |
| Results | 11 | State the actual number of raters and subjects/objects which were included and the number of replicate observations which were conducted. | 9 |
|  | 12 | Describe the sample characteristics of raters and subjects (e.g. training, experience). | 9, table 1 |
|  | 13 | Report estimates of reliability and agreement including measures of statistical uncertainty. | 10, table 4 |
| Discussion | 14 | Discuss the practical relevance of results. | 13, 14 |
| Auxiliary material | 15 | Provide detailed results if possible (e.g. online). | Tables 1-4 |
